# Discovering endometriosis biomarkers with multiplex cytokine arrays

**DOI:** 10.1186/s12014-019-9248-y

**Published:** 2019-07-11

**Authors:** Bao Weisheng, Ceana H. Nezhat, Gordon F. Huang, Ying-Qing Mao, Neil Sidell, Ruo-Pan Huang

**Affiliations:** 1 RayBiotech, Inc, 3607 Parkway Lane, Peachtree Corners, GA 30092 USA; 2 Nezhat Medical Center, 5555 Peachtree Dunwoody Rd #276, Atlanta, GA 30342 USA; 3 Emory University, 201 Dowman Dr, Atlanta, GA 30322 USA; 4 Guangzhou RayBiotech, 79 Ruihe Road, Huangpu District, Guangzhou, 510630 China; 5 Affiliated Cancer Hospital and Institute of Guangzhou Medical University, Guangzhou Medical University, No. 232 Waihuan Dong Rd, Guangzhou University Town, Panyu District, Guangzhou, 510006 China; 6 South China Biochip Research Center, 79 Ruihe Road, Huangpu District, Guangzhou, 510630 China

**Keywords:** Arrays, Biomarkers, Cytokines, Endometriosis, Multiplex array

## Abstract

**Background:**

Chronic pelvic pain is often overlooked during primary examinations because of the numerous causes of such “vague” symptoms. However, this pain can often mask endometriosis, a smoldering disease that is not easily identified as a cause of the problem. As such, endometriosis has been shown to be a potentially long-term and often undiagnosed disease due to its vague symptoms and lack of any non-invasive testing technique. Only after more severe symptoms arise (severe pelvic pain, excessive vaginal bleeding, or infertility) is the disease finally uncovered by the attending physician. Due to the nature and complexity of endometriosis, high throughput approaches for investigating changes in protein levels may be useful for elucidating novel biomarkers of the disease and to provide clues to help understand its development and progression.

**Methods:**

A large multiplex cytokine array which detects the expression levels of 260 proteins including cytokines, chemokines, growth factors, adhesion molecules, angiogenesis factors and other was used to probe biomarkers in plasma samples from endometriosis patients with the intent of detecting and/or understanding the cause of this disease. The protein levels were then analyzed using K-nearest neighbor and split-point score analysis.

**Results:**

This technique identified a 14-marker cytokine profile with the area under the curve of 0.874 under a confidence interval of 0.81–0.94. Our training set further validated the panel for significance, specificity, and sensitivity to the disease samples.

**Conclusions:**

These findings show the utility and reliability of multiplex arrays in deciphering new biomarker panels for disease detection and may offer clues for understanding this mysterious disease.

**Electronic supplementary material:**

The online version of this article (10.1186/s12014-019-9248-y) contains supplementary material, which is available to authorized users.

## Background

Endometriosis is an enigmatic disease in which endometrial tissue is found outside the uterine cavity. This disease affects roughly 10% of all reproductive-aged women and is a complex syndrome consisting of multiple vague symptoms such as pelvic pain and infertility. As many as 70% of women with infertility or chronic pelvic pain are affected, and yet the cause(s) of this disease is still unknown [1, 2]. The disease is often masked by its generalized symptoms and is either undiagnosed or misdiagnosed in a majority of patients until more severe symptoms arise or surgical detection methods are pursued. To this end, there is currently a lack of non-surgical diagnostic tests that show adequate sensitivity or specificity to be useful for diagnostic purposes. Thus, laparoscopic inspection with corresponding histological analysis is currently required for accurate diagnosis. This lack of any noninvasive diagnostic test, such as MRI or CT imaging to detect endometriotic lesions frequently results in suboptimal care of at-risk patients.

Although the histological *sine qua non* of endometriosis includes the presence of endometrial cells in extrauterine sites, research over the past decade has provided strong evidence that the intrauterine environment in these women is also affected [3–6]. As a result, methods for detecting consistent changes that occur in the eutopic endometrium as well as from non-invasive sources from women with endometriosis have become a subject of intense investigation. However, even with recent findings, the current field of testing options remains extremely limited. Left without noninvasive diagnostic tests, full diagnosis of endometriosis continues to require costly invasive surgery, most commonly performed via laparoscopy, to document the presence of visible endometriotic lesions. The average cost of these techniques in the U.S. approaches $5000 and it has been estimated that when both direct and indirect expenses are considered, endometriosis diagnostics and patient care account for up to $22 billion in U.S. healthcare costs per year [7]. Due to the costly nature and general risks involved with surgical procedures, many clinicians elect to forgo surgical confirmation of endometriosis, particularly in adolescents and young women, leaving potential diseased patients undiagnosed [8, 9]. Hence, an alternative to laparoscopy is desperately needed to facilitate earlier and accurate diagnosis of patient’s disease, or at least serve to reduce the number of patients who warrant surgical procedures [10].

Recent advances in antibody microarray technologies have been a boon for the identification and detection of disease biomarkers in cancer, immunologic disease, and neurological impairments [11–13]. Antibody array platforms can be used for most liquid sample types, and can easily screen patient samples in a high-throughput manner. Using this technology, uncovering some of the underlying markers indicative of endometriosis could help in our understanding of the development, presence, and treatment of this disease. To this end, such tools could allow for a future non-invasive test on at-risk patients using samples as simple as urine, serum, or plasma, thereby lowering the financial and surgical burden for those suffering from other causes of pelvic pain and infertility. However, current disease markers for endometriosis are limited, creating a need for further characterization and identification of potential biomarkers that could prove useful from an understanding of the disease as well as diagnostic standpoint. In this manuscript, we report the utility of using antibody array platforms to screen endometriosis confirmed patient samples for potential disease biosignatures as a preliminary buildout for understanding this disease. This technology probed a large subset of potential biomarkers to uncover a panel of analytes that are suggestive of endometriosis disease. Subsequently, the reproducibility of the findings was validated with the creation of a custom built biomarker array specific for these identified analytes. Such a platform has the potential to fulfill the need of offering a low cost, simplified, high throughput method for disease biomarker discovery, as well as for the development of diagnostic platforms.

## Materials and methods

### Sample collection

Human plasma samples from 70 endometriosis patients, 5 polycystic ovarian syndrome patients, 6 pelvic adhesion patients, 15 ovarian cyst patients, and 52 healthy controls included in the study were collected from the affiliated hospitals, Emory University and Northside Hospital. Patient selection and collection protocols were approved by the Institutional Review Boards of the Emory University School of Medicine (IRB000002405) and Northside Hospital (Atlanta, GA). Patients were stratified by age, disease state, and menstrual cycle if possible. Written consent was obtained when collecting samples from both patients and healthy controls.

### Multiplex array technology

Quantitative sandwich-based antibody arrays (RayBio^®^ Human Cytokine Array G-Series) were developed as 6 distinct arrays (Human Inflammation Array Q3, Human Growth Factor Array Q1, Human Chemokine Array Q1, Human Receptor Array Q1, Human Cytokine Array Q4, Human Cytokine Array G6), each representing a unique set of 40–60 antigen-specific antibodies to detect a total of 260 markers on a glass slide matrix (Additional file 1: Table S1). Glass slides were printed as 16 identical subarrays consisting of spots of each antigen-specific capture antibody for that array. Printed slides were placed in chamber assemblies to allow for incubation of each subarray with a different sample. After blocking each subarray with a blocking buffer, subarrays were incubated with plasma samples and antigen standards. Following extensive washing to remove non-specific binding, the cocktail of biotinylated detection antibodies was added to the arrays. After extensive washing, the array slides were incubated with a streptavidin conjugated Cy3 compatible dye (Anaspec, Fremont, CA). The fluorescent signals were then obtained using a laser scanner system (GenePix 4000 BA, Molecular Devices LLC, Sunnyvale, CA). To increase the accuracy of the measurement, two to four replicates per antibody were spotted, and the averages of the median signal intensities across replicate spots (minus local background) were used for all calculations. With this technique, the coefficient of variation (CV) remains around 10% or less for all arrays.

### Multiplex array repeatability

To test our multiplex array reproducibility, we built a custom fully quantitative array for these defined targets (Quantibody Array). After the training set of 122 samples was completed, a logistic regression model was generated based on the selected marker panel with the highest performance. A blind testing of four endometriosis samples and two control samples from the training set was then built. Those six blind samples would be predicted as control or endometriosis following the Logreg model.

### Data analysis

A non-parametric Mann–Whitney U test was used to test the significance in protein expression levels between endometriosis and healthy control groups. *P* values less than 0.05 were considered to be statistically significant. To determine the signal threshold, signals from the arrays were measured in the absence of samples (using blocking buffer as a blank) and repeated 10 times. The signals generated using blanks were averaged and the standard deviation (SD) was calculated. Signals with values lower than the average blank signal + 2 × SD were considered as background. The data was analyzed by split-point score analysis (SSA). The split point divides the sample space into two intervals, one for endometriosis and one for normal controls. The best split point score of each marker was chosen to ensure the minimization of misclassified samples. For each marker, a score of 0 was assigned to a sample if it fell in the normal control interval for that marker; a score of 1 was assigned to a sample if it fell in the endometriosis interval. Overall, an individual was assigned a score as the sum of these assigned scores for N different markers. Therefore, the range of such score was between 0 to N. A given threshold (T) was chosen to optimally separate endometriosis from healthy controls, i.e. a given individual with a total score < T is predicted to have normal status, whereas an individual with a total score > T was judged as endometriosis. A non-parametric K-nearest neighbor analysis (KNN) was used to determine the specificity, sensitivity, and accuracy of the 14 endometriosis-specific biomarker panel based on 5 neighbors (*k*) and Euclidean distance.

## Results

Since endometriosis is thought to have an underlying inflammatory basis, we hypothesized that aberrant levels of cytokines or similar molecules could be detected in diseased patient plasma. Thus, we began our study by probing disease patient samples with one of our large multiplex arrays. This array can identify levels of 260 proteins including inflammatory, chemotactic, and growth factors in an effort to uncover biomarkers indicative of endometriosis disease. This multiplex array served to both limit potential marker bias, while also allowing for as large a breadth as possible for disease detection. In order to identify potential differences with sufficient specificity, sensitivity, and accuracy, we enrolled a test patient group consisting of 70 medically diagnosed endometriosis patients as well as 52 healthy controls who were confirmed to be disease-free if there was no evidence of endometriosis following laparoscopic examination by an experienced gynecologic surgeon. The general characteristics of all the 122 patients can be seen in Table 1. The diagnosed patients had undergone standard laparoscopic surgeries to confirm endometriotic lesions outside the uterus.

**Table 1:** Clinical characteristics of study population

	Control	Endometriosis
Total patients	52	70
*Age (years)*		
Mean (SD)	40.3 (6.0)	36.1 (7.1)
Median (range)	41 (25–52)	35 (20–49)
*Patient cycle*		
Menstrual (n)	8	8
Luteal (n)	13	20
Follicular (n)	13	20
Data unavailable (n)	20	22

With our initial probe of the 122 patient samples, our multiplex arrays identified 38 cytokines that were significantly altered between diseased and healthy patients; 21 of which showed increased expression in endometriosis samples, and 17 of which showed decreased expression (Table 2). Interleukins IL-6, IL-7, IL-8, IL-12p70, and IL-15 were all significantly upregulated in endometriotic patients. This would support a generalized inflammatory state present at the site of endometrial lesions, and potentially a global increased inflammatory state as well. The immunologic response hypothesis is further supported by the increase in other inflammatory and chemotactic markers like MCP-1, TNF-β, I-309 (CCL1), IFN-γ, and I-TAC (CXCL11), and Eotaxin (CCL11) in diseased patients. Alongside these apparent inflammatory increases, there were also changes in the presence or absence of several cell surface adhesion molecules. CEACAM-1 was significantly upregulated in the disease patient samples, while CD14, EpCAM, and NrCAM were decreased when compared to healthy patients. Such a surface receptor change may indicate the needed trafficking changes that are required for immunological access to the diseased tissue. Such changes may also allow recruitment of accessory cells that facilitate the expansion of the endometrial tissue outside of its normal boundaries. Or, the changes could directly be involved in the escape of endometrial cells into the surrounding tissues by means of changes in cell adhesion moieties. Lastly, growth and angiogenic factors like Angiopoietin 1 (ANG-1), Angiostatin, Lipocalin-2, and ErbB3 were decreased in endometriosis patients, while IGF-1 was significantly elevated in diseased patients. This suggests that increases in blood vessel growth are either not required for endometrial tissue expansion, or by the time the tissue escapes, such factors are no longer required to support the extrauterine tissue.

**Table 2:** Significantly altered proteins between disease and healthy patients

	*p* value	Fold change
6Ckine	0.034	0.52
ANG-L	0.042	0.80
Angiostatin	0.002	0.59
BLC	0.038	1.36
CD14	0.000	0.86
CD40	0.023	0.72
CEACAM-1	0.006	1.23
Cripto-1	0.027	0.99
DAN	0.015	1.92
DKK-1	0.001	0.63
E-Cadherin	0.004	0.51
ENA-78	0.005	0.63
Eotaxin	0.000	1.62
EpCAM	0.003	0.55
ERBB3	0.03	0.80
Fc-γ RIIB/C	0.012	1.11
Follistatin	0.031	0.70
I-309	0.02	1.81
IFN-γ	0.043	1.23
IGF-1	0.027	5.79
IGFBP-3	0.036	1.12
IGFBP-4	0.016	1.97
IL-12P70	0.034	2.40
IL-13 R1	0.039	4.24
IL-15	0.017	1.25
IL-6	0.017	1.19
IL-7	0.016	1.20
IL-8	0.008	1.17
l-TAC	0.003	1.43
LAP	0.001	0.67
Lipocalin-2	0.006	0.92
MCP-1	0.011	1.20
NRCAM	0.041	0.70
RAGE	0.011	1.59
TARC	0.006	0.36
TIMP-1	0.043	0.97
TNF-β	0.021	1.32
VEGF-D	0.02	3.46

*P* value: Mann–whitney U test, *P* < 0.05; n = 52 controls, 70 endometriosis

Given these 38 cytokine differences, we next sought to determine if any of the cytokine perturbations were sufficient to specifically identify endometriosis patients from healthy controls, either alone, or in combination with other markers. To this end we ran a split-score analysis to identify overlapping biomarkers that specifically identify endometriosis patient samples. This analysis identified a unique 14 panel biomarker subset among the altered cytokine profile that could differentiate between endometriosis and normal patient samples (Table 3). These biomarkers included 6Ckine, CD14, CEACAM-1, ENA-78, ERBB3, IL-7, I-TAC, LAP (TGF-β), Lipocalin-2, MCP-1, NrCAM, RAGE, TARC, and TNF-β. When evaluating these 14 markers, we noted that they were not all associated with one common process or pathway, but instead spanned across multiple pathways from inflammation, to angiogenesis, to cellular growth factors. Such a finding supports a multifactorial disease etiology that may require a methodology to identify multiple rather than single biomarkers for disease detection. Such multiple cytokine biomarkers, while of obvious interest from a diagnostic perspective, could also provide some interesting insight into the cause and development of this disease.

**Table 3:** 14 marker panel list

6Ckine
CD14
CEACAM-1
ENA-78
ERBB3
IL-7
I-TAC
LAP (TGF-b)
Lipocalin-2
MCP-1
NrCAM
RAGE
TARC
TNF-β

**Fig. 1:**
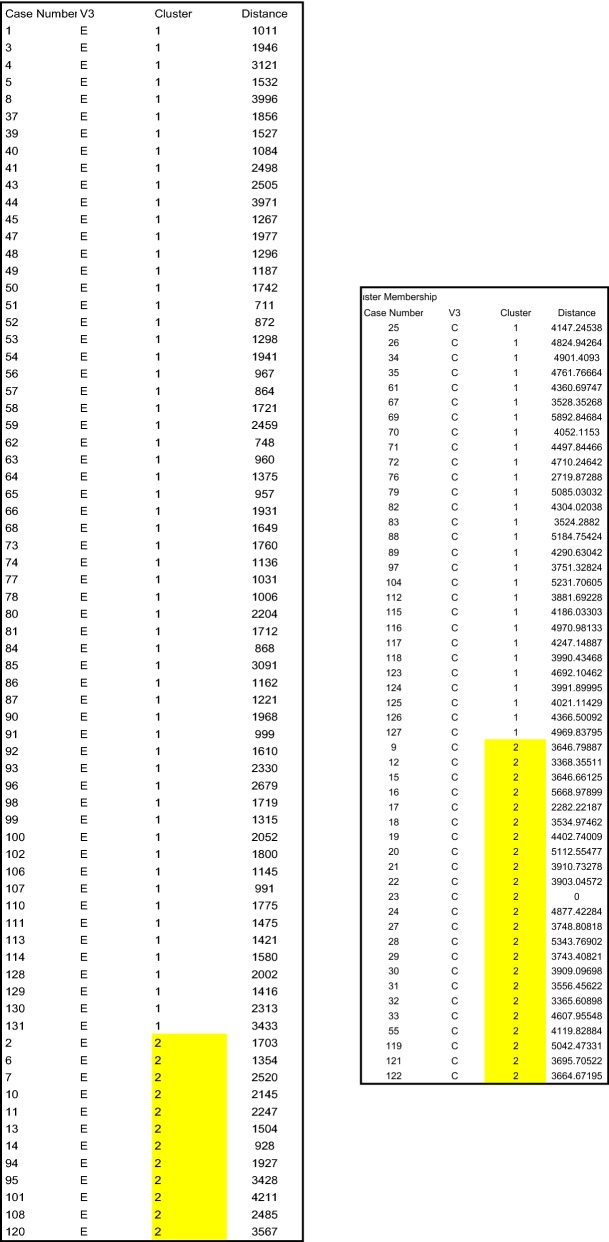
K-nearest neighbor analysis of 14 protein biomarker panel comparing endometriosis and healthy controls. The sensitivity, specificity and accuracy were 82.8%, 48.1% and 68.0%, respectively

While initial findings of a biomarker panel from a broad array is of interest, we needed to further the data set with K-nearest neighbor (KNN) analysis to better support our findings. To evaluate our systems compatibility and reliability, we set out to determine the specificity, sensitivity, and accuracy of the smaller panel. To this end, we profiled the 14 panel array by KNN analysis. KNN analysis showed that our biomarker panel had a sensitivity of 82.8%, specificity of 48.1%, and accuracy of 68.0% in detecting endometriosis (Fig. 1).

**Fig. 2:**
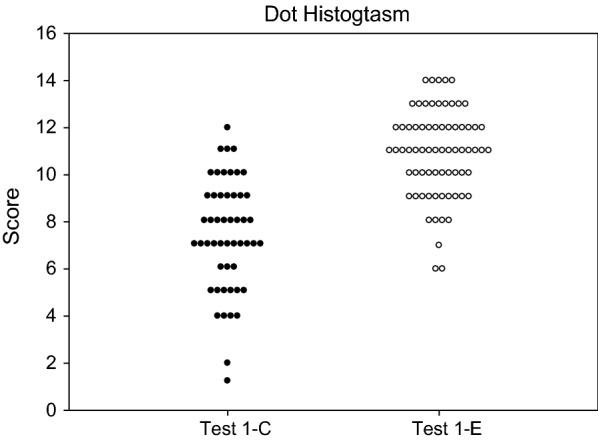
Dot histogram plot of our 14-marker panel split-point score classification of plasma from healthy control (n = 52) and endometriosis (n = 70). A cutoff score ≥ 9 was set. Samples from endometriosis patients should have a score ≥ 9, whereas normal plasma samples should have a score < 9. Based on the score cutoff, the sensitivity, specificity and accuracy were 90%, 67.3% and 80.3%, respectively

Given the fair specificity of the assay by KNN analysis, we also set out to run addition empirical testing to further examine our biomarker panel. This same panel was then tested by split score analysis (SSA), wherein if the sample had a biomarker value in the endometriosis range, it received a score of 1 for that marker, while if it was in the normal range it received a score of 0. Using a cutoff score of 9, we were able to generate a split point analysis sensitivity value of 90%, specificity of 67.3%, and an accuracy of 80.3% in identifying the disease patient samples (Fig. 2).

**Fig. 3:**
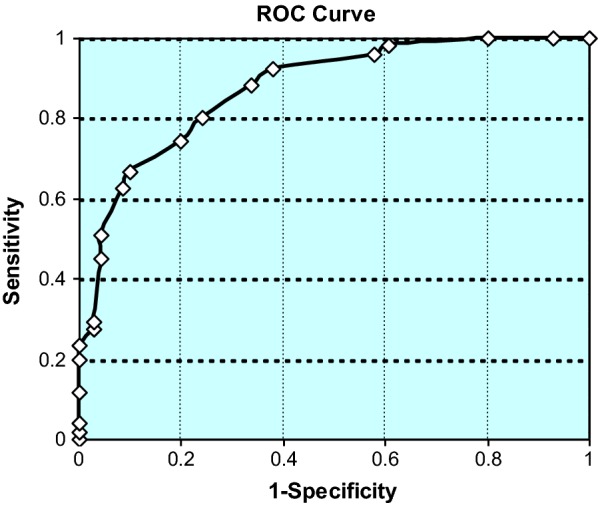
Receiver operating characteristic (ROC) curve for 14-marker panel logistic regression scores in 122 sample data set of endometriosis and healthy control groups. The area under ROC curve (AUC) was 0.874, and its 95% CI was 0.81–0.94

We ran a receiver operating characteristic (ROC) analysis of the 14 marker panel, and found that the area under the curve (AUC) was 0.874, with a confidence interval of 0.81–0.94, suggesting high specificity for the detection of diseased samples when compared to controls (Fig. 3). When combined together, the three analysis techniques show 68%, 80.3%, and 87.4% correct identification rates of diseased samples (an average of 78%), and offer good support for the overall panel in plasma-based disease detection. Additionally, the values for sensitivity and specificity were also very promising for our panel.

Finally, we set out to ensure the reliability and reproducibility of our array testing methodology, as well as to add a layer of data that could be useful for future testing. To test for reproducibility, we developed a customized Quantibody array, which used the same 14 antibody pairs from the original array. We chose 6 patient samples at random from the training set and then analyzed the samples using the 14 biomarker Quantibody array. This customized 14 analyte panel with our 1.982 logit cutoff allowed us to accurately identify all 6 of the samples correctly with regards to their disease state (Table 4).

**Table 4:** Multiplexed quantitative antibody array repeatability

Sample code	Logit	Prediction	True diagnosis
NS76	7.860	E	E
NS62	2.744	E	E
NS74	2.450	E	E
NS101	2.154	E	E
NS65	1.760	C	C
NS054	− 0.386	C	C

Logit cutoff = 1.982; *E* endometriosis, *C* control

When the Logit cutoff was 1.982, 14 marker panel can give an overall 87.5% accuracy in the training set. If Logit was bigger than 1.982, the sample was predicted as endometriosis; Otherwise, it was predicted as control. The results from Table 1 showed all the six samples were predicted correctly; which strongly demonstrated the Quantibody array reliability and repeatability

To rule out the possibility that the 14 potential biomarkers identified in this study (6Ckine, CD14, CEACAM-1, ENA-78, ERBB3, IL-7, I-TAC, LAP (TGF-β), Lipocalin-2, MCP-1, NrCAM, RAGE, TARC, TNF-β) are nonspecific to other inflammatory gynecological conditions, we also compared the differential expression of the 14 proteins between 52 healthy controls with 5 polycystic ovarian syndrome patients (PCOS), 6 pelvic adhesion patients, and 15 ovarian cyst patients. Volcano plot analyses of the 14 proteins demonstrate that seven proteins may be unique to endometriosis. (Additional file 2: Tables S2–S5, Figures S1–S4). Nine of the proteins were identified as being statistically significant between the endometriosis patients and healthy controls with a *p* value < 0.05 [6Ckine, CD14, ENA-78, I-TAC, LAP (TGF-β), NrCAM, RAGE, TARC, TNF-β]. Only 1 protein, Lipocalin-2 or LAP, was differentially expressed in PCOS or pelvic adhesion patients, respectively, compared to healthy controls. Three proteins (6Ckine, Lipocalin-2, IL-7), on the other hand, were differentially expressed between ovarian cyst patients and healthy controls.

Together, this data suggests an excellent biomarker panel for detecting endometriosis disease, independent of any histological or other detection techniques. Our automatable and high throughput methodology could allow for cheaper initial detection techniques, as well as reasons to explore potential non-invasive biomarkers of disease. Additionally, our study confirms the reliability of larger multiplex array type assays in biomarker discovery, and also demonstrates the utility of going from a broad screen to a smaller more targeted screen. With no loss in reliability and reproducibility, this could bring biomarker testing techniques to the forefront of the field, as smaller target arrays are more economical, high throughput, and less sample dependent.

## Discussion

Endometriosis remains a silent disease as its symptoms are vague and are often ignored by both patients and physicians. This disease is an extreme burden to child-bearing-aged and older women, and untreated patients are at risk of more severe negative pathologies. Because in many cases the mild symptoms alone do not justify an invasive procedure such as laparoscopic surgery, at-risk patients are often not fully screened for this disease, delaying the time of initial diagnosis. As such, other non-invasive techniques need to be a priority for the field and justify further exploration. However, for a non-invasive test to be developed, more understanding of the etiology and the biomarkers of the disease need to be evaluated. Ideally, a non-invasive test could be built into normal blood workups during a patient’s annual checkup and help identify potentially at risk patients suffering from abdominal related symptoms. At the very least, such tests may be able to eliminate those patients for which invasive surgeries are not warranted based on biomarker workups.

With the goal of uncovering biomarkers indicative of endometriosis, we opted for a large multiplex antibody array that could simultaneously probe patient samples for 260 proteins. This large and non-biased approach identified 38 potentially important proteins as being altered during endometriosis disease (or at least associated with the presence of disease in patient samples). Interestingly, these proteins are involved in inflammation, cellular growth, chemotaxis, and angiogenesis, suggesting involvement of multiple pathways during endometriosis development, symptoms, or disease progression. While 38 proteins may have been significantly altered by the underlying disease, we identified a 14-panel target set that had a unique specificity for endometriosis within our cohort of 70 endometriosis patients and 52 healthy controls.

The markers of endometriosis that were differentially regulated covered multiple potential disease facets. A number of innate and adaptive chemoattractant molecules were significantly different between healthy and diseased patients. Notably, innate cellular chemoattractants (IL-12, I-309/CCL1, Eotaxin, MCP-1, IL-6, IL-8, and IFN-γ) were almost exclusively elevated in endometriosis patients with the sole exception of the neutrophil chemoattractant ENA-78/CXCL5. This finding highlights the potential smoldering inflammation present at the sites of endometrial lesions, where a constant warning milieu of cytokines are being secreted to continue the supply of innate immune cells. Adaptive T and B cell markers also saw some changes related to various chemokines involved in development and/or recruitment. Interestingly, consistent with a mild inflammation status, common T_H_1 cell inflammatory chemokines were more likely to be elevated (IFNγ, IL-12, and IL-6), while other T cell attractants involved in T_H_2 promotion were decreased in endometriosis patients (TARC and 6Ckine, respectively). In support of these findings, it has been suggested in a number of studies that endometriosis is more linked to a T_H_1 polarization than a T_H_2 polarization [14–16].

A number of cellular adhesion molecules and receptors were shown to be differentially regulated. The mechanisms behind these changes remain unclear, but given the presence of extrauterine tissue, and potential inflammatory events underlying it, these alterations could reflect global shifts in immune cell trafficking in response to the disease [17]. Increased soluble and surface CEACAM-1 has been associated with endometrial tumors, suggesting that the body responds to both the extrauterine tissue and endometrial tumors in a similar fashion [18]. It is interesting that the levels of NrCAM were reduced in patient plasma, while previously this protein has been shown to be upregulated in extrauterine tissues [19]. Given few examples of its expression at the site of disease, the mechanism by which NrCAM is secreted into the circulation remains unclear. Similar unexpected results were found for EpCAM which has also been shown to be increased in extrauterine tissues, while we saw a corresponding drop in the plasma of our endometriosis patients [20].

It is also worth noting that the samples employed in this biomarker screening study consisted of 70 endometriosis patients and 52 healthy controls. The identification of differentially-expressed proteins between these two groups with one sample set can lead to overfitting of the data. In order to minimize overfitting, we employed three analysis models, k nearest neighbor, split score, and ROC analysis, which resulted in 68–87.4% correct identification rates of diseased samples. We also tested 6 samples at random using an array printed at a different time than the array used for the initial study; 4 diseased samples and 2 healthy control samples were accurately characterized. Furthermore, we determined that 7 of the 14 biomarkers were unique to endometriosis when we compared the differential protein expression between healthy controls and patients with polycystic ovarian syndrome, pelvic adhesion, or ovarian cysts.

To support this determination of biomarker specificity, we also searched Pubmed for publications related to the 14 cytokines and disorders that incur gynecological inflammation, including “endometriosis,” “pelvic inflammatory disease,” “polycystic ovarian syndrome,” “pelvic adhesion,” “sexually transmitted disease,” “uterine fibroid,” “uterine cancer,” and “ovarian cancer.” Two of the proteins, CD14 and TNF-β, have been previously associated with all the conditions in at least 2 publications and an overall average of ~ 90 publications (Additional file 3: Table S6). While these biomarkers are likely related to inflammation rather than endometriosis, other proteins in our 14-cytokine panel may be more specific to endometriosis. For example, less than three publications have linked ITAC and NrCAM with a gynecological disorder other than endometriosis, and ENA78 has not been identified in any gynecological disease, including endometriosis. To our knowledge, this is the first time that five cytokines, CEACAM1, ENA78, ITAC, Lipocalin-2, and NrCAM, have been identified as potential biomarkers of endometriosis. Clearly, validation of the 14-cytokine panel using a larger independent cohort including patients with other gynecological disorders is necessary.

As the utility of single biomarkers to diagnose or prognosticate specific diseases is rapidly being shown to be untenable, there is a greater appreciation for the role of multiple proteins or factors in the deciphering and determination of certain conditions. This is especially true when the etiology and symptoms of the disease are masked. Recent biomarker studies utilize multiplex platforms capable of screening tens to thousands of markers simultaneously, helping to make the most out of every drop of precious sample. These approaches also benefit from their general breadth, and unbiased approach. From Kawasaki’s disease, to aortic aneurysms, to rheumatoid arthritis, many recent studies have used these platforms to discover both single markers of disease, as well as to identify pathways and multiple involved proteins [21–25]. Further analysis of global biomarker changes may help identify new targets for disease diagnosis, new underlying mechanisms behind disease development, and potentially help outline new targeted therapies. While additional analysis of our 14 biomarker panel is needed to validate their utility in the diagnosis of endometriosis, the protocols and techniques used here support the use of multiplex analysis in revealing unknown disease signatures from a global proteomic view. We hope these findings support the future analysis of endometriosis and other disease samples with multiplex technologies, leading ultimately to new disease biomarkers, a greater understanding of pathways involved in disease, and ultimately new and better treatments for at risk and diseased patients.

## Conclusions

Using a fully quantitative multiplex cytokine array, we probed for the presence of 260 cytokines, chemokines, and growth factors to identify a panel of biomarkers for endometriosis disease. Differential expression of 14 cytokines in serum distinguished endometriosis patients from healthy controls, with seven proteins not differentially expressed in patients with other inflammatory gynecological disorders like PCOS, ovarian cysts, and pelvic adhesions. Our training set further validated the panel for significance, specificity, and sensitivity to the disease samples. While further testing needs to be done using an independent cohort to fully validate the panel, our findings show the utility of multiplex arrays in deciphering new biomarker panels for detecting disease using noninvasive sample types.

## Additional files

**Additional file 1.** Supplemental Table S1.

**Additional file 2.** Supplemental Tables S2–S5, Figures S1–S4.

**Additional file 3.** Supplemental Table S6.

## Data Availability

The datasets used and/or analysed during the current study are study are either included in this published article or available from the corresponding author on reasonable request

## Abbreviations

ANG-1: angiopoietin 1
AUC: area under the curve
CCL11: eotaxin, C–C motif chemokine ligand 11
CD14: CD14 molecule
CEACAM-1: carcinoembryonic antigen-related cell adhesion molecule 1
ENA-78/CXCL5: C–X–C motif chemokine ligand 5
EpCAM: epithelial cell adhesion molecule
I-309/CCL1: C–C motif chemokine ligand 1
IFN-γ: interferon gamma
IGF-1: insulin-like growth factor I
IL-6: interleukin 6
IL-8: interleukin 8
IL-12: interleukin 12
KNN: k nearest neighbor
MCP-1: monocyte chemotactic protein-1
NNK: neural network analysis
NrCAM: neuronal cell adhesion molecule
ROC: receiver operating characteristic
SSA: split-point score analysis

## References

[1] Mihalyi A, Gevaert O, Kyama CM, Simsa P, Pochet N, De Smet F (2010). Non-invasive diagnosis of endometriosis based on a combined analysis of six plasma biomarkers. Hum Reprod.

[2] Kennedy S, Bergqvist A, Chapron C, D’Hooghe T, Dunselman G, Greb R (2005). ESHRE guideline for the diagnosis and treatment of endometriosis. Hum Reprod.

[3] Smarr MM, Kannan K, BuckLouis GM (2016). Endocrine disrupting chemicals and endometriosis. Fertil Steril.

[4] Rier SE, Martin DC, Bowman RE, Dmowski WP, Becker JL (1993). Endometriosis in rhesus monkeys (*Macaca mulatta*) following chronic exposure to 2,3,7,8-tetrachlorodibenzo-p-dioxin. Fundam Appl Toxicol.

[5] Rier SE (2008). Environmental immune disruption: a comorbidity factor for reproduction?. Fertil Steril.

[6] Zeitoun KM, Bulun SE (1999). Aromatase: a key molecule in the pathophysiology of endometriosis and a therapeutic target. Fertil Steril.

[7] Soliman AM, Yang H, Du EX, Kelley C, Winkel C (2016). The direct and indirect costs associated with endometriosis: a systematic literature review. Hum Reprod.

[8] Parasar P, Ozcan P, Terry KL (2017). Endometriosis: epidemiology, diagnosis and clinical management. Curr Obstet Gynecol Rep.

[9] Stuparich MA, Donnellan NM, Sanfilippo JS (2017). Endometriosis in the adolescent patient. Semin Reprod Med.

[10] Taylor HS, Adamson GD, Diamond MP, Goldstein SR, Horne AW, Missmer SA (2018). An evidence-based approach to assessing surgical versus clinical diagnosis of symptomatic endometriosis. Int J Gynaecol Obstet.

[11] Jayanthi V, Das AB, Saxena U (2017). Recent advances in biosensor development for the detection of cancer biomarkers. Biosens Bioelectron.

[12] Duarte JG, Blackburn JM (2017). Advances in the development of human protein microarrays. Expert Rev Proteomics..

[13] Schwartzbaum J, Wang M, Root E, Pietrzak M, Rempala GA, Huang RP (2017). A nested case–control study of 277 prediagnostic serum cytokines and glioma. PLoS ONE.

[14] Podgaec S, Dias Junior JA, Chapron C, Oliveira RM, Baracat EC, Abrão MS (2010). Th1 and Th2 immune responses related to pelvic endometriosis. Rev Assoc Med Bras (1992).

[15] Szymanowski K, Niepsuj-Biniaś J, Dera-Szymanowska A, Wołuń-Cholewa M, Yantczenko A, Florek E (2013). An influence of immunomodulation on Th1 and Th2 immune response in endometriosis in an animal model. Biomed Res Int.

[16] Podgaec S, Abrao MS, Dias JA, Rizzo LV, de Oliveira RM, Baracat EC (2007). Endometriosis: an inflammatory disease with a Th2 immune response component. Hum Reprod.

[17] Mosbah A, Nabiel Y, Khashaba E (2016). Interleukin-6, intracellular adhesion molecule-1, and glycodelin A levels in serum and peritoneal fluid as biomarkers for endometriosis. Int J Gynaecol Obstet.

[18] Fischer C, Drillich M, Odau S, Heuwieser W, Einspanier R, Gabler C (2010). Selected pro-inflammatory factor transcripts in bovine endometrial epithelial cells are regulated during the oestrous cycle and elevated in case of subclinical or clinical endometritis. Reprod Fertil Dev.

[19] Khan MA, Sengupta J, Mittal S, Ghosh D (2012). Genome-wide expressions in autologous eutopic and ectopic endometrium of fertile women with endometriosis. Reprod Biol Endocrinol.

[20] Van den Berg LL, Crane LM, van Oosten M, van Dam GM, Simons AH, Hofker HS (2013). Analysis of biomarker expression in severe endometriosis and determination of possibilities for targeted intraoperative imaging. Int J Gynaecol Obstet.

[21] Ko TM, Kuo HC, Chang JS, Chen SP, Liu YM, Chen HW (2015). CXCL10/IP-10 is a biomarker and mediator for Kawasaki disease. Circ Res.

[22] Ramos-Mozo P, Rodriguez C, Pastor-Vargas C, Blanco-Colio LM, Martinez-Gonzalez J, Meilhac O (2012). Plasma profiling by a protein array approach identifies IGFBP-1 as a novel biomarker of abdominal aortic aneurysm. Atherosclerosis.

[23] Ramírez J, Ruíz-Esquide V, Pomés I, Celis R, Cuervo A, Hernández MV (2014). Patients with rheumatoid arthritis in clinical remission and ultrasound-defined active synovitis exhibit higher disease activity and increased serum levels of angiogenic biomarkers. Arthritis Res Ther.

[24] Zhou Q, Mao YQ, Jiang WD, Chen YR, Huang RY, Zhou XB (2012). Development of IGF signaling antibody arrays for the identification of hepatocellular carcinoma biomarkers. PLoS ONE.

[25] Patel CG, Yee AJ, Scullen TA, Nemani N, Santo L, Richardson PG (2014). Biomarkers of bone remodeling in multiple myeloma patients to tailor bisphosphonate therapy. Clin Cancer Res.

