# Supplementary material for: Discovering endometriosis biomarkers with multiplex cytokine arrays
Source: Clin Proteomics. 2019 Jul 11;16:28. doi: 10.1186/s12014-019-9248-y (PMC6621950; doi:10.1186/s12014-019-9248-y)
Supplement: Supplementary file 2 — Additional file 2. Supplemental Tables S2–S5, Figures S1–S4. [file 12014_2019_9248_MOESM2_ESM.docx]

**Additional Files**

**Table S2. Comparison of 14 biomarkers between 70 endometriosis patients and 52 healthy controls**


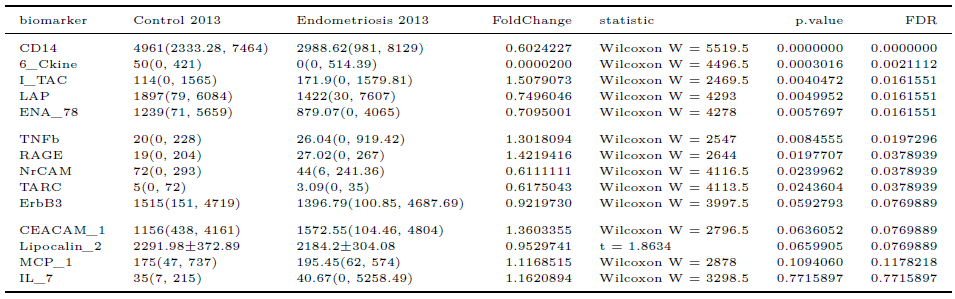


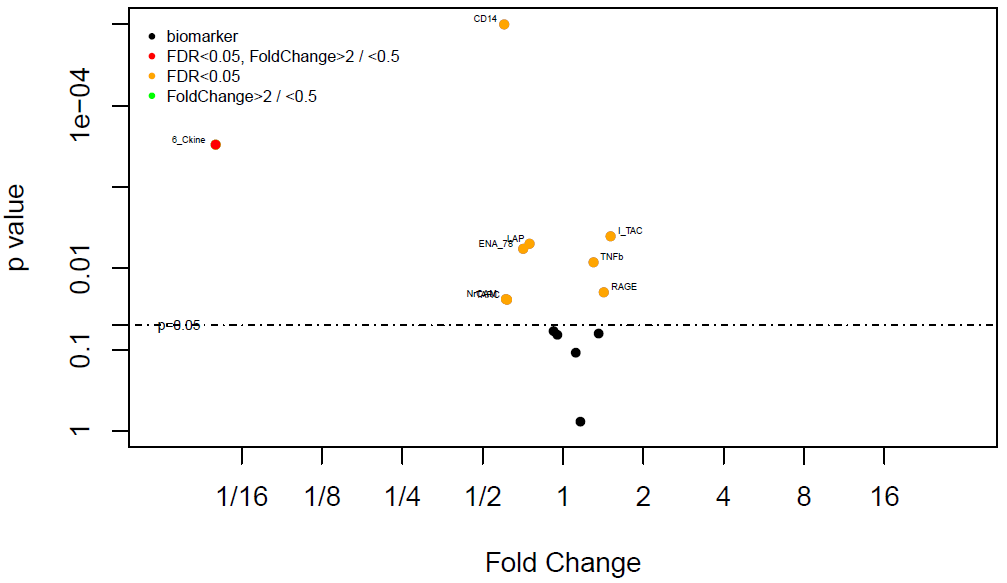
**Figure S1. Volcano plot comparing the fold change and p-value of 14 biomarkers of 70 endometriosis patients and 52 healthy controls in which each point represents a biomarker.**

**Table S3. Comparison of 14 biomarkers between 15 ovarian cyst patients and 52 healthy controls**

**
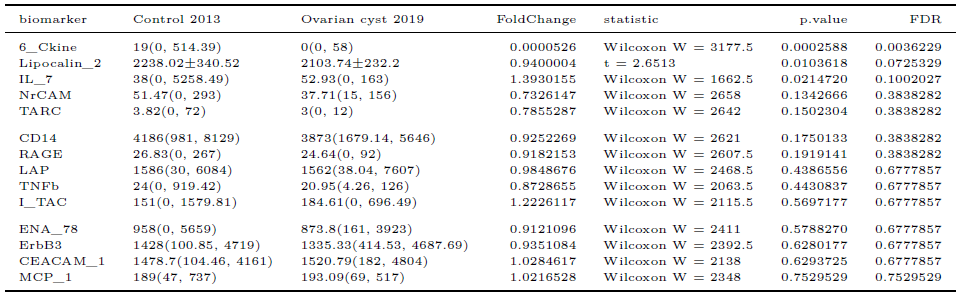
**

**
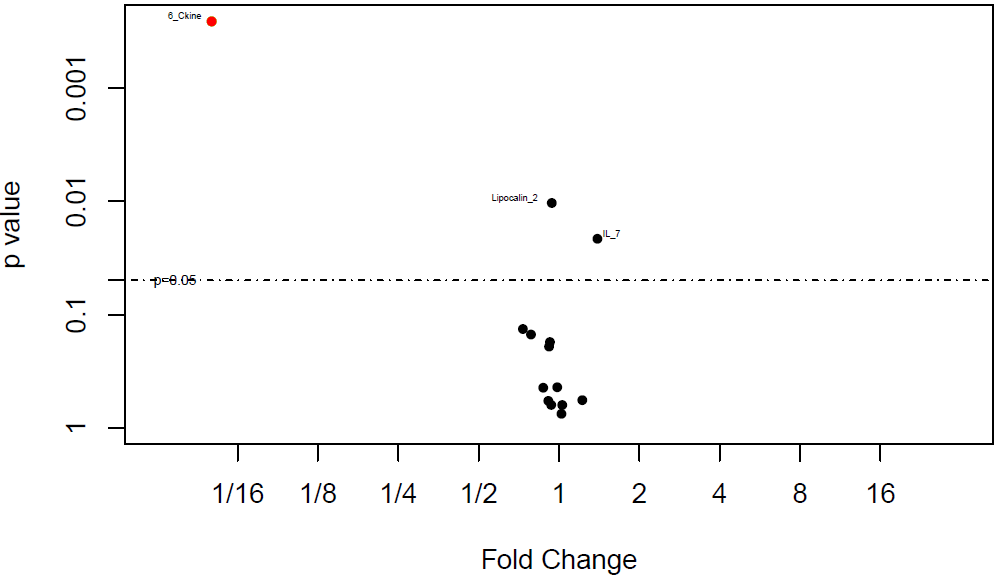
Figure S2. Volcano plot comparing the fold change and p-value of 14 biomarkers of 15 ovarian cyst patients and 52 healthy controls in which each point represents a biomarker.**

**Table S4. Comparison of 14 biomarkers between 6 pelvic adhesion patients and 52 healthy controls**

**
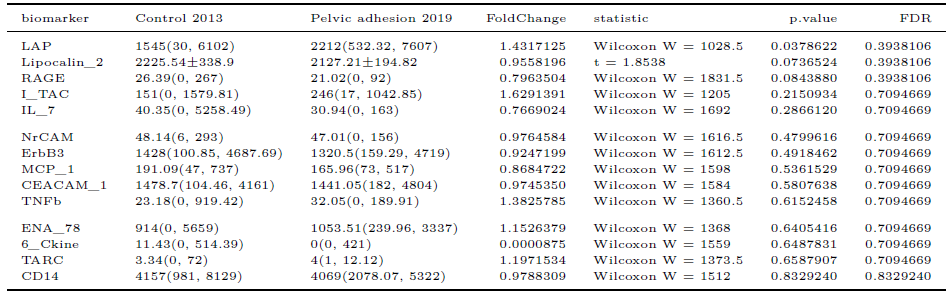
**

**
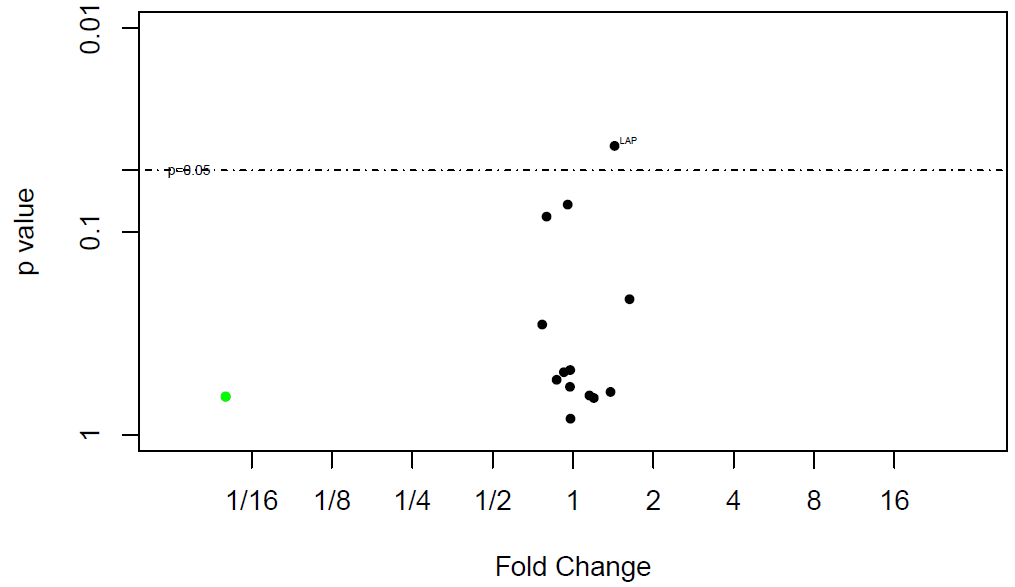
**

**Figure S3. Volcano plot comparing the fold change and p-value of 14 biomarkers of 6 pelvic adhesion patients and 52 healthy controls in which each point represents a biomarker.**

**Table S5. Comparison of 14 biomarkers between 5 PCOS patients and 52 healthy controls**

**
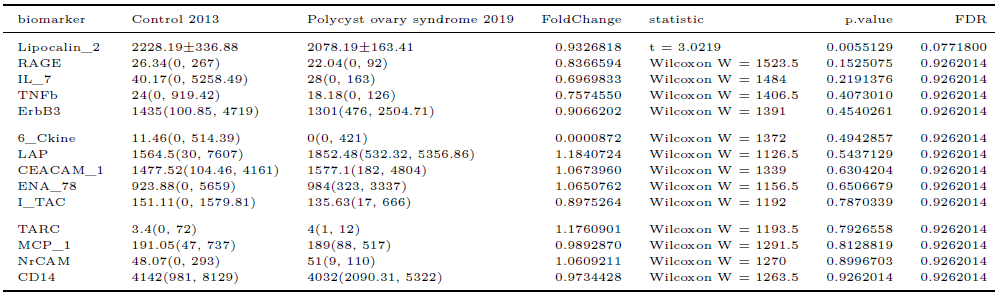
**

**
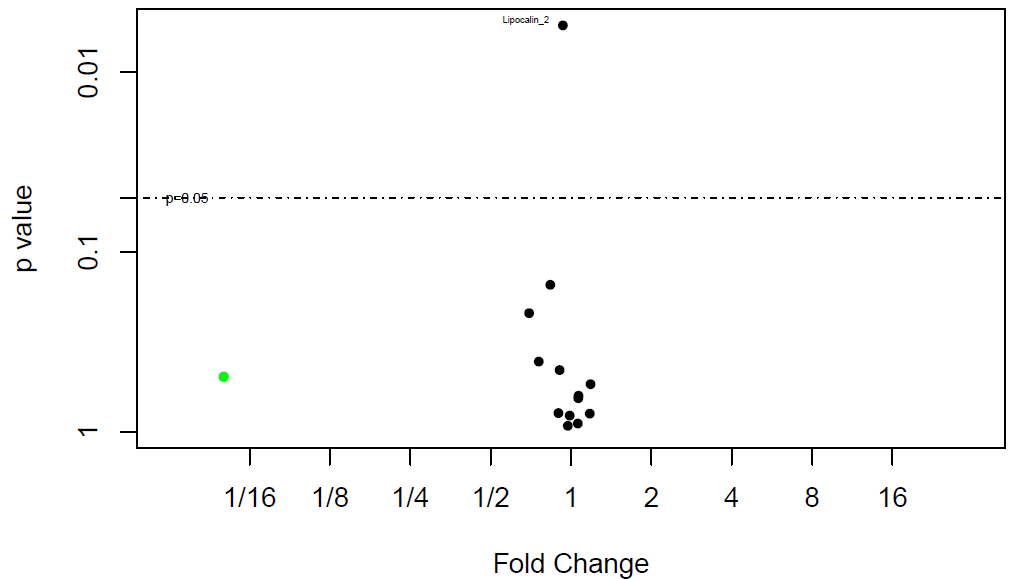
**

**Figure S4. Volcano plot comparing the fold change and p-value of 14 biomarkers of 5 PCOS patients and 52 healthy controls in which each point represents a biomarker.**
